# Application of Three Ecological Assessment Tools in Examining Chromatographic Methods for the Green Analysis of a Mixture of Dopamine, Serotonin, Glutamate and GABA: A Comparative Study

**DOI:** 10.3390/molecules26185436

**Published:** 2021-09-07

**Authors:** Atiah H. Almalki, Ibrahim A. Naguib, Fahad S. Alshehri, Badrah S. Alghamdi, Hashem O. Alsaab, Yusuf S. Althobaiti, Sameer Alshehri, Fatma F. Abdallah

**Affiliations:** 1 Department of Pharmaceutical Chemistry, College of Pharmacy, Taif University, P.O. Box 11099, Taif 21944, Saudi Arabia; i.abdelaal@tu.edu.sa; 2 Addiction and Neuroscience Research Unit, Taif University, P.O. Box 11099, Taif 21944, Saudi Arabia; h.alsaab@tu.edu.sa (H.O.A.); ys.althobaiti@tu.edu.sa (Y.S.A.); 3 Department of Pharmacology and Toxicology, College of Pharmacy, Umm Al-Qura University, Makkah 21955, Saudi Arabia; fsshehri@uqu.edu.sa; 4 Neuroscience Unit, Department of Physiology, Faculty of Medicine, King Abdulaziz University, Jeddah 22252, Saudi Arabia; basalghamdi@kau.edu.sa; 5 Pre-Clinical Research Unit, King Fahad Medical Research Center, King Abdulaziz University, Jeddah 22252, Saudi Arabia; 6 Department of Pharmaceutics and Pharmaceutical Technology, College of Pharmacy, Taif University, P.O. Box 11099, Taif 21944, Saudi Arabia; s.alshehri@tu.edu.sa; 7 Department of Pharmacology and Toxicology, College of Pharmacy, Taif University, P.O. Box 11099, Taif 21944, Saudi Arabia; 8 Pharmaceutical Analytical Chemistry Department, Faculty of Pharmacy, Beni-Suef University, Beni-Suef 62514, Egypt; dr.zahrafared@yahoo.com

**Keywords:** dopamine, serotonin, glutamate, GABA, NEMI, GAPI, ESA, green chemistry

## Abstract

The assessment of greenness of analytical protocols is of great importance now to preserve the environment. Some studies have analyzed either only the neurotransmitters, dopamine, serotonin, glutamate, and gamma-aminobutyric acid (GABA), together or with other neurotransmitters and biomarkers. However, these methods have not been investigated for their greenness and were not compared with each other to find the optimum one. Therefore, this study aims to compare seven published chromatographic methods that analyzed the four neurotransmitters and their mixtures using the National Environmental Method Index, Analytical Eco-Scale Assessment (ESA), and Green Analytical Procedure Index (GAPI). As these methods cover both qualitative and quantitative aspects, they offer better transparency. Overall, GAPI showed maximum greenness throughout the analysis. Method 6 was proven to be the method of choice for analyzing the mixture, owing to its greenness, according to NEMI, ESA, and GAPI. Additionally, method 6 has a wide scope of application (13 components can be analyzed), high sensitivity (low LOQ values), and fast analysis (low retention times, especially for glutamate and GABA).

## 1. Introduction

Neurotransmitters carry electrochemical signals and act as messengers between neurons and their target cells. As such, they are very important for normal brain function [1]. Their dysfunction causes several mental disorders, such as neurodegeneration, depression, schizophrenia, stress, and addiction [2,3]. Analysis of these neurotransmitters helps understand the changes in their concentration at the molecular level and their impact on brain function.

Dopamine is a catecholamine involved in regulating circulatory, motor, and cognitive functions, along with reward-seeking behavior [4]. It has been reported to be, in part, responsible for various neurological conditions, such as Parkinson’s disease [5], schizophrenia, and psychosis, as well as addictions [6]. Dopamine deficiency is believed to cause motor and cognitive dysfunction in patients diagnosed with PD [7,8,9,10], while high dopamine levels have been reported in cases of psychosis and schizophrenia [11].

Glutamate is the main excitatory neurotransmitter in the mammalian central nervous system (CNS) [12,13]. It is known to mediate sensory information and motor coordination, as well as cognitive and memory functions [14,15,16]. Several neurodegenerative diseases, including Alzheimer’s disease (AD) [17,18,19], multiple sclerosis (MS) [20], amyotrophic lateral sclerosis (ALS), and Huntington’s disease [21,22], have been linked to glutamate excitotoxicity. Thus, many approaches have been suggested to modulate glutamate release and prevent excitotoxicity in many brain disorders.

Serotonin (5-hydroxytryptamine; 5-HT) is a monoamine neurotransmitter that has a wide range of regulatory effects on the CNS, blood pressure, heart rate, and coagulation activity [23,24]. It has been implicated in numerous diseases, such as cardiac arrhythmia and hypertension, along with multiple neurological disorders, such as migraine, epilepsy, depression, anxiety, ALS, attention-deficit hyperactivity disorder (ADHD), autism spectrum disorder [25], schizophrenia, obsessive-compulsive disorder, addiction, PD, and MS [26,27,28,29,30,31,32,33,34,35,36,37,38].

Gamma-aminobutyric acid (GABA) is an inhibitory neurotransmitter regulated by neurons and glial cells. GABAergic inhibitory effects can be either phasic or tonic [39]. GABAergic system dysfunction, due to altered levels of GABA, mutation in GABA receptors, or changes in the GABA receptor density, results in a wide range of neurological disorders, such as ADHD, Autism spectrum disorder [25], epilepsy, depression, anxiety, PD, MS, and AD [10,40,41,42,43,44,45,46].

Simultaneous analysis of these neurotransmitters will help determine their roles in the pathophysiology of neurological disorders. However, analytical procedures require the use of reagents which can produce toxic residues that are hazardous to the operators and cause environmental contamination. The chemical industry expels approximately 5 billion tons of harmful chemical wastes into the environment every year. Additionally, more than 300 billion dollars are spent annually for the treatment, control, and elimination of these chemical wastes. Various synonyms, such as clean chemistry and sustainable chemistry are used to refer to chemistry that involves analytical processes that do not adversely affect the environment. The concept of green chemistry, which was defined by P.T. Anastas as “a set of principles that reduces or eliminates the use or generation of hazardous substances in the design, manufacture, and application of chemical products” [47], emerged in the early 1990s. Green chemistry is based on 12 principles suggested by Anastas and Warner in 1998 [47]: waste prevention, atom economy, less hazardous chemical synthesis, designing safer chemicals, safer solvents and auxiliaries, design for energy efficiency, use of renewable feedstock, reduced derivatives, use of catalysis, design for degradation, real-time analysis for pollution prevention, and inherently safer chemistry for accident prevention.

Three major tools were selected for assessing the greenness of the analytical methods in this study: (1) the National Environmental Method Index [48,49], characterized by its simplicity, where one fast look at the pictogram will be enough to have general information about the environmental impact of a procedure and which provides general information about the greenness of the method; (2) the Analytical Eco-Scale assessment (ESA) [50,51,52], a semi-qualitative method which provides eco-scale analysis of the analytical method, based on the calculation of a numerical score to get a final value indicating the greenness of the method, where 100 is the ideal green procedure.; and (3) the Green Analytical Procedure Index (GAPI) [53], a new tool which can evaluate the green character of an entire analytical methodology, from sample collection to final determination, so as to provide specific information related to the greenness of each analytical method.

The analytical abstracts service provided by the Royal Society of Chemistry (http://pubs.rsc.org/lus/analytical-abstracts, accessed on 15 December 2020) was utilized to obtain information about all the methods used for the analysis of quaternary mixtures of neurotransmitters. The results yielded eight chromatographic methods for the analysis of different neurotransmitters simultaneously. Three studies reported quantification of six neurotransmitters using liquid chromatography with tandem mass spectrometry (LC-MS/MS) [54,55,56]. Another study detected seven neurotransmitters with the help of microdialysis coupled with hydrophilic interaction chromatography–tandem mass spectrometry (MD-HILIC–MS/MS) [57]. In the other studies, eight neurotransmitters were studied using LC-MS [58,59]. Only one study reported quantification of 10 neurotransmitters and their metabolites [60]. All these methods were used for the quantification of Dopamine, Glutamate, GABA, and 5-hydroxytryptamine (5-HT) and its metabolites.

The aim of this work is to experimentally evaluate the greenness of seven chromatographic methods used for the analysis of a quaternary neurotransmitter mixture containing dopamine, serotonin, glutamate, and GABA. It also aims to compare these methods and determine the fastest and most sensitive one with the widest scope of application. This study will lay a foundation for further research in this field.

## 2. Results and Discussion

### 2.1. Method Implementation and Limitations

#### 2.1.1. National Environmental Method Index

The National Environmental Method Index (NEMI) has one of the largest databases of environmental analytical methods developed by the Methods and Data Comparability Board [48]. This database is freely available for scientists at www.nemi.gov (accessed on 20 January 2021) and includes links to different guidelines, summaries, and numerous complete methods. The profiles and acceptance criteria of greenness are developed and optimized by interpreting the data from an analytical procedure into a greenness report that includes chemicals used, pH, and waste generated. The report patterns are distinguished by four features: hazardous; corrosive; persistent, bio-accumulative, and toxic (PBT); and waste; all are represented by quarters in a circle and each quarter is colored, either less green or greener [49].

**Figure 1:**
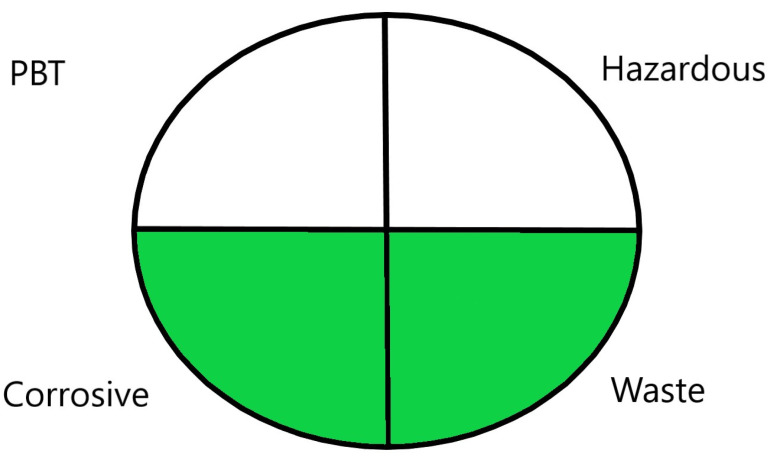
NEMI evaluation pictogram results for all evaluated chromatographic methods.

Upon investigation of all seven evaluated chromatographic methods using the NEMI, all methods (Figure 1) provided acceptable waste and corrosive profile but were found to be hazardous and not fulfilling the PBT criteria. These results showed that NEMI is not enough for detailed comparison, and another assessment method is needed for effective comparison.

#### 2.1.2. Analytical Eco-Scale Assessment (ESA)

The ESA method involves quantitative assessment of the analytical method, and is used to compare different analytical methods for evaluation of greenness. It displays the amount of material being consumed and the waste generated during the experiment.

This assessment tool is used to illustrate the degree of greenness of the analytical method. It is composed of 100 points, where 100 represents a perfect level of greenness, with no penalty points. The penalty point decreases the total score of greenness and indicates the damaging effect of the hazardous materials that are being utilized in the analytical method. A method is considered green if the score is more than 75%; acceptable if the score is between 50% and 75%; and least green if the score is less than 50% [50,51,52].

One drawback of this method is that no clear details are provided regarding the problems and undesirable impact of the harmful material. It only gives a number that represents the entire result [53]. Accordingly, ESA cannot be used as the only benchmark for determining the greenness of a method, when comparing two or more methods.

Upon application of ESA as an assessment tool, most of the methods provided close results of around 80; however, they could still be differentiated. As per Table 1, method 4 (by Xiaozhe Zhang et al. [56]) was the most non-green with a score of 71, while method 1a (by Tae-Hyun Kim et al.) was the most green with a score of 90. However, method 1a had a major problem of not analyzing the quaternary neurotransmitters under investigation together. This issue is addressed later in this paper.

#### 2.1.3. Green Analytical Procedure Index (GAPI)

This method was suggested by J. Płotka-Wasylka and involves evaluation of the entire analytical procedure/protocol [53]. It comprises three steps of analytical process description and has five pentagrams for evaluating and quantifying the whole process. The first step describes the sample collection of every analytical procedure; the second step describes protection of the sample from potential physical and chemical changes; the last step determines and quantifies the analyses. GAPI is a tool that displays the greenness of each step as a pictogram. The colors used in this method are green, yellow, and red, where green represents maximum greenness and red denotes minimum greenness [53]. Table 2 describes the application of the GAPI method to each of the seven chromatographic methods under investigation.

### 2.2. Comparison of the Three Assessment Methods and Nominating the Most Green Chromatographic Method

The development and evolution of the scientific literature related to the assessment of green analytical methods is growing annually, due to the need to advance new practices that comply with ecological requirements [61,62,63,64,65,66,67,68]. The emergence of many greenness assessment studies has gained attention as they are intended to reduce waste production, energy consumption, and cost and generate eco-friendly methods.

The NEMI method produced the same figure (Figure 1) for greenness assessment of the seven chromatographic methods analyzing the four neurotransmitters (Dopamine, Serotonin, Glutamate, and GABA) (Table 1), with only two green quarters for all methods. This method was not efficient at differentiating greenness assessments when compared with the GAPI and ESA methods. Therefore, NEMI should be coupled with more detailed assessment tools for full-spectrum evaluation of greenness.

Methods 1a and 1b were proposed by (Tae-Hyun Kim et al. [47]), wherein method 1a offers analysis of BH4 and DA together, and method 1b offers analysis of 5-HT, NE, EP, Glu, and GABA together. Neither method offers simultaneous analysis of the four targeted neurotransmitters, although they were mentioned in the same research article, and therefore, it will be logical to exclude them from the comparison.

Method 6 (proposed by Hua-Lin Cai et al. [60]) exhibited most greenness based on GAPI (Table 2) with no significant difference from method 5 (by Kevin Y. Zhu et al. [55]) and method 2 (by Ya-Bin Tang et al. [57]), based on the ESA score (method 2 scored 88, method 5 scored 85, and method 6 scored 84). Method 6 can be described as the most green, considering all stages of the analysis process. Additionally, another advantage offered by method 6 is the simultaneous analysis of ten neurotransmitters compared to only six by method 5 and seven neurotransmitters by method 2, thus granting higher relative selectivity for method 6.

Considering the greenness and cost effectiveness, method 6 is the method of choice for analysis.

In other terms of comparison, methods 1 (1a and 1b) and 2 were isocratic, while all other methods offered gradient elution of the mobile phase, indicating that they could be simpler in application. Method 1 was the fastest method of analysis for dopamine, which eluted at 0.84 min, and serotonin, which eluted at 1.72 min. However, glutamate eluted at 1.52 min in methods 2 and 6, whereas GABA eluted at 1.36 min in method 6. Method 6 provided an edge by offering optimum analysis time for glutamate and GABA. Furthermore, method 6 offered analysis of the most complicated matrix (13 components) and therefore possessed the widest scope of application compared to all other methods under study.

Additional advantage of method 6 was that it had the lowest limit of quantification [69] value for all neurotransmitters (therefore offering the highest sensitivity, where all the values were expressed as pM/mL.

## 3. Methods

### 3.1. Greenness Assessment Protocols

The lack of methods that are capable of evaluating the greenness of different analytical processes is one of the main issues when determining the greenness of a method. It is vital to obtain a suitable, ideal, and straightforward method to assess the greenness (qualitatively and quantitatively) of an analytical method. This helps analytical chemists to choose a method that is eco-friendly, based on scientific evaluation. Three greenness assessment methods were applied in our study. The principles behind these methods are summarized below:

#### 3.1.1. National Environmental Methods Index Label

NEMI is the oldest assessment tool that has been used to evaluate the greenness of analytical methods. In this method, a circle that is divided into quarters is drawn (Pictogram symbols); each quarter embodies one feature that might have a possible harmful ecological impact [48]. If the method aligns with the codes of green chemistry, all the quarters are colored green. However, if a given quarter does not comply with any one of the NEMI conditions, it will not be colored.

This procedure will not fulfill the greenness conditions if any of the reagents or substances used in the analytical method is listed in the toxic release inventory or in the Resource Conservation and Recovery Act. Usually, analytical chemists are advised not to use any of the chemicals that are persistent, bio-accumulative, or toxic; they are told to keep the pH above 2 and below 12 during analysis to prevent an extremely corrosive ecosystem throughout the analysis. In addition, they are advised to retain any generated waste that is <50 g.

NEMI has many advantages over other methods, including straightforwardness of the assessment and uncomplicated interpretation of the score. In day-to-day life, a pictogram that represents the greenness of a method will be enough to have a decent opinion about the ecological influence of the analytical procedure [53]. Nonetheless, this method has a major drawback, as the amount of chemicals used in the analysis and waste produced are not considered, either qualitatively or quantitatively. In addition, the assessment of each chemical or reagent that had been used in the procedure in official lists is difficult and time consuming. Overall, this assessment method is useful for creating a general idea about the greenness of an analytical method.

#### 3.1.2. Analytical Eco-Scale Method

The analytical eco-scale assessment method was proposed by Van Aken et al., [70]. This method performs analyses based on a numerical score, where 100 represents the greenest. The negative impact of chemicals that are being used in the procedure is represented by penalty points. These points are subtracted from the analytical Eco-Scale (100) if hazardous substances, waste production, or high energy utilization show a negative impact on the ecological system and depart from the ideal green method. As the impact of hazardous chemical materials depends on their quantity, the total penalty points should be determined by multiplying the sub-total penalty points by the given amount of hazardous substance. To assess the risk of the reagents used in the analytical procedures (the basis for penalty points), different sets of dangerous materials can be applied. The Globally Harmonized System of Classification and Labeling of Chemicals [71], which is the most comprehensive and updated classification of chemicals, is usually applied to evaluate the extent of ecological, physical, or health hazards caused by these substances [71].

The total penalty points of the entire method should be included in the Eco-Scale calculation. This method is considered green if the Eco-Scale is above 75 points; acceptable green analysis if the scale is between 50 and 75 points; and inadequate green analysis if the Eco-Scale is below 50 points. The penalty points are estimated by pictograms and signal words. Each substance can be distinguished by one or more of the nine pictograms (flame, flame over circle, corrosion, gas cylinder, skull, crossbones, exclamation mark, environment, and health hazard), and penalty points are allocated for each substance pictogram. Importantly, none of the pictograms is represented by zero penalty points. In addition, there are two signal words to describe the hazardous material in the GHS–danger and warning. The less hazardous substance (“warning” pictogram) is equal to one penalty point while the highly hazardous substance (“danger” pictogram) is equal to two penalty points. This system can be applied to calculate the penalty points of hazards [52].

There are many advantages of using the Eco-Scale method in assessing the greenness of analytical methods. This method has the ability to analyze the amount of chemical substances and waste production semi-quantitatively. It is not as complicated when compared to the GAPI method and has distinct assessment criteria. This method demonstrates the impact of analytical procedures on the ecological system in a more quantitative way than the NEMI method. Moreover, it offers the ease of comparison of different analytical procedures with different aspects of ecological impact in its assessment. However, one main drawback of this procedure is that the final outcome does not indicate the source of the undesirable ecological impact [72].

#### 3.1.3. Green Analytical Procedure Index (GAPI)

**Figure 2:**
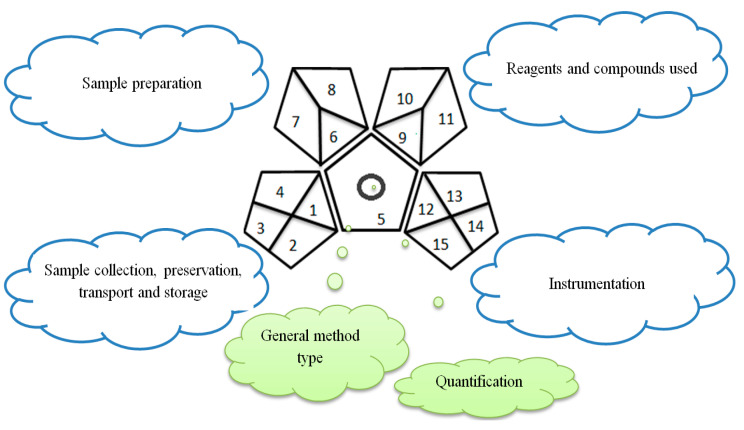
Green analytical procedure index pictogram with description.

This method was established to assess the greenness of each phase of an analytical method by applying pictograms and using a color scale, with three levels of assessment for each phase (Figure 2).

In GAPI assessments, a definite symbol with five pentagrams is employed to evaluate an analytical method. The color scale which represents the ecological impact of material in each step of the methodology ranges from green (the best) through yellow (medium) to red (the worst). Each area reflects a distinct feature of the assessed analytical method. The area is filled with green color if the conditions are accomplished or filled with red color if conditions are not achieved. The graphic presentation of the evaluation tools allows investigators to arrive at conclusions about the various green measures. Hence, this greenness assessment tool is very valuable for comparing different methods. GAPI clearly and evidently indicates the weakest points in analytical procedures. Nevertheless, it does not provide numerical evaluation, unlike the ESA method, which makes it less favorable to analysts, yet it offers complete qualitative evaluation of all the analytical protocol stages.

## 4. Conclusions

Assessment of greenness of analytical methods is gaining high visibility and interest among analysts. This study helped compare the greenness of seven chromatographic methods used for simultaneous analysis of dopamine, serotonin, glutamate, and GABA. Application of all the three methods of assessment, NEMI, ESA, and GAPI, together helped create better transparency of both qualitative and quantitative aspects of greenness.

Based on the results of this study, it is very clear that method 6 is the best to analyze the quaternary neurotransmitter mixture, whether for its greenness (according to NEMI, ESA, and GAPI), the scope of application (13 components can be analyzed), sensitivity (low LOQ values), or time of analysis (low retention times, especially for glutamate and GABA).

## Figures and Tables

**Table 1:** ESA, NEMI, and GAPI tools for assessment of greenness values of different analytical methods described in the literature for determination of the studied neurotransmitters.

Method No.	Employed Instrument and Chromatographic Settings	Eco-Scale Analytical Assessment Method		NEMI Method	GAPI Method
		Penalty Points	Analytical Eco-Scale Score		
**1a**	Analytical Method by Tae-Hyun Kim et al. [54]: Chromatography technique: LC that coupled with electrospray tandem MS No. of analytes: 2 analytes including (dopamine and tetrahydrobiopterin) Stationary phase: Sepax Polar-Imidazole (2.1 × 100 mm, i.d., 3 μm) column Mobile phase: *Isocratic,* 10 mM NH_4_HCO_2_ in CH_3_CN/H_2_O (75:25, *v*/*v*, 300 μL/min) Flow rate: 0.3 mL min^−1^ Time of analysis: 5 min Biological sample: mouse brain tissue	CH_3_CN 4 H_2_O 0 NH_4_HCO_2_ 1 Energy consumption 2 Waste production 3 Occupational Risk 0 Penalty points = 10	**90**	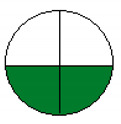	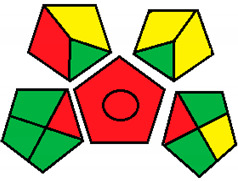
**1b**	Analytical method by Tae-Hyun Kim et al. [54]: Chromatography technique: LC-MS No. of analytes: 5 analytes including (NE, serotonin, EPI, GABA, Glu) Stationary phase: Luna 3 μ C18 (3.0 × 150 mm, i.d., 3 μm) column. Mobile phase: *Isocratic,* 1% CH_2_O_2_ in CH_3_CN/H_2_O (20:80, *v*/*v*). Flow rate: 0.35 mL min^−1^. Time of analysis: 5 min. Biological sample: mouse brain tissue	CH_2_O_2_ 6 CH_3_CN 4 H_2_O 0 Energy consumption 2 Waste production 3 Occupational risk 0 Penalty points = 15	**85**	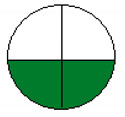	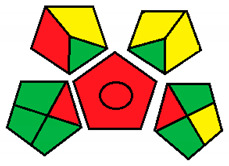
**2**	Analytical Method by Ya-Bin Tang et al. [57]: Chromatography technique: microdialysis coupled with HICMS No. of analytes: 7 analytes (Ach, serotonin, DA, NE, Glu, GABA, and glycine) were quantified using Stationary phase: Merck ZIC-HILIC column (2.1 mm–100 mm, 3 mm; Merck Sequant, Umea, Sweden). Mobile phase: *Isocratic,* CH_3_OH and H_2_O (55:45, *v*/*v*), 20 mM NH_4_HCO_2_ and adjusted to pH 3.0 with CH_2_O_2_). Flow rate: 0.2 mL min^−1^. Time of analysis: within 5 min. Biological sample: embryonal carcinoma stem cells	H_2_O 0 CH_3_OH 6 NH_4_HCO_2_ 1 CH_2_O_2_ 6 Energy consumption 2 Waste production 3 Occupational risk 0 Penalty points = 18	**88**	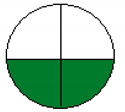	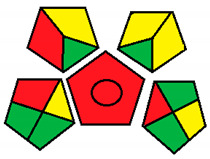
**3**	Analytical method by Qianqian Wang et al. [58]: Chromatography technique: microdialysis coupled with LC-MS No. of analytes: 8 (Glu, ASP, GABA, Ser, Tau, Ach, DA, and 5-HT) Stationary phase: C_18_ Mobile phase: *Isocratic,* Water (0.12% formic acid): Methanol (0.06% formic acid), (67:33, *v*/*v*). Flow rate: 0.5 mL min^−1^ Time of analysis: 19.5 min. Biological sample: rat brain tissue	H_2_O 0 CH_3_OH 6 CH_2_O_2_ 6 Energy consumption 2 Waste production 5 Occupational risk 0 Penalty points = 19	**81**	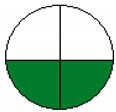	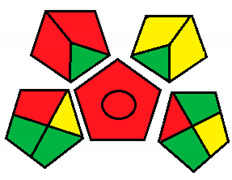
**4**	Analytical method by Xiaozhe Zhang et al. [56]: Chromatography technique: HIC-MS simultaneously in primate cerebral cortex No. of analytes: 6 analytes including (acetylcholine, serotonin, dopamine, GABA, glutamate and aspartate) Stationary phase: fused-silica capillary Mobile phase: *Gradient,* (0.00 to 6.00 min, 85% B to 45% B, then 45% B was kept for 8 min), (A) H_2_O solution containing 20 mM NH_3_HCO_2_ and 1% formic acid, (B) was CH_3_CN. Flow rate: 3.5 mL min^−1^. Time of analysis: 14 min. Biological sample: cerebral cortex	CH_3_CN 8 NH_4_HCO_2_ 2 CH_2_O_2_ 12 Energy consumption 2 Waste production 5 Occupational risk 0 Penalty points = 29	**71**	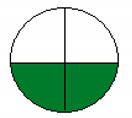	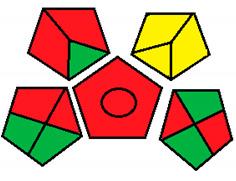
**5**	Analytical method by Kevin Y. Zhu et al. [55]: Chromatography technique: LC-MS No. of analytes: 6 analytes including (GABA, dopamine, EPI, NE, Glu, and serotonin) Stationary phase: C_18_ column (3.0 µm i.d., 100 × 2.1 mm). Mobile phase: *Gradient,* of 0.1% formic acid in water (A) and 0.1% formic acid in acetonitrile (B) using the following gradient program: 0–2 min, isocratic gradient 1.0% (B); 2–6 min, linear gradient 1.0%–90.0% (B); 6–10 min, isocratic gradient 90.0% (B). Flow rate: 0.2 mL min^−1^ Time of analysis: 10 min Biological sample: rat brain tissue	CH_3_CN 4 CH_2_O_2_ 6 Energy consumption 2 Waste production 3 Occupational risk 0 Penalty points = 15	**85**	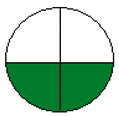	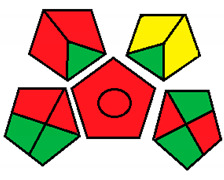
**6**	Analytical method by Hua-Lin Cai et al. [60]: Chromatography technique: LC-MS No. of analytes: 8 analytes including (HVA, NE, VMA, MHPG, 5-HT, 5-HIAA, Glu, and GABA) in human plasma. Stationary phase: C_18_ column (3 µm, 150 × 2 mm) Mobile phase: *gradient;* (A) H_2_O with 20 mM NH_4_CH_3_CO_2_ and 0.1% CH_2_O_2_, and (B) CH_3_CN. Flow rate: 0.250 mL min^−1^. Time of analysis: 27.1 min. Biological sample: human plasma	NH_4_CH_3_CO_2_ 1 CH_3_CN 4 CH_2_O_2_ 6 Energy consumption 2 Waste production 3 Occupational risk 0 Penalty points = 16	**84**	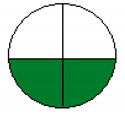	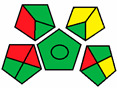
**7**	Analytical method by Linjia Sun et al. [59]: Chromatography technique: liquid chromatography-tandem mass spectrometry (LC-MS/MS) method No. of analytes: 8 analytes including (Glu, GABA, Ach, 5-HT, DA, NE, Trp, and Tyr) Stationary phase: C18 MG column (4.6 × 150 mm, 5 mm). Mobile phase: gradient; (A) H_2_O containing 0.1% CH_2_O_2_, and solvent (B) CH_3_CN. Flow rate: 0.8 mL min^−1^. Time of analysis: 15 min. Biological sample: rat brain tissue	CH_3_CN 8 CH_2_O_2_ 12 Energy consumption 2 Waste production 5 Occupational risk 0 Penalty points = 27	**73**	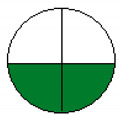	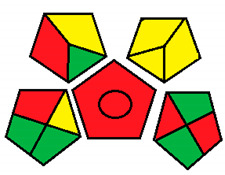

**Table 2:** Detailed descriptions of Green Analytical Procedure Index (GAPI) parameters for analytical methods of assessment of the studied neurotransmitters.

Method No.	Category	Method	Figure
**1a**	Sample preparation Collection (1) Preservation Transport (3) Storage (4) Method category: direct or indirect (5) Scale of extraction (6) Solvents/reagents used (7) Extra treatments (8) Reagent and Solvents Amount (9) Health risk (10) Safety risk (11) Instrumentation Energy consumption (12) Occupational risk (13) Waste (14) Waste treatment (15)	Offline (red) None (green) None (green) None (green) Extraction required (red) Nano-extraction (green) Non-green solvents and reagents (red) Simple treatment (yellow) 10< mL (green) NFPA = 2, moderate toxicity (yellow) NFPA = 3, high flammability (yellow) >1.5 Kwh per sample (red) Hermetic sealing of analytical process (green) 1–10 mL (yellow) Recycling possible (green)	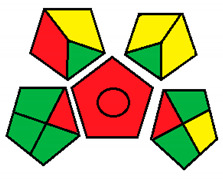
**1b**	Sample preparation Collection (1) Preservation Transport (3) Storage (4) Method category: direct or indirect (5) Scale of extraction (6) Solvents/reagents used (7) Extra treatments (8) Reagent and Solvents Amount (9) Health risk (10) Safety risk (11) Instrumentation Energy consumption (12) Occupational risk (13) Waste (14) Waste treatment (15)	[34] Offline (red) None (green) None (green) None (green) Extraction required (red) Nano-extraction (green) Non-green solvents and reagents (red) Simple treatment (yellow) 10< mL (green) NFPA = 2, moderate toxicity (yellow) NFPA = 3, high flammability (yellow) >1.5 Kwh per sample (red) Hermetic sealing of analytical process (green) 1–10 mL (yellow) Recycling possible (green)	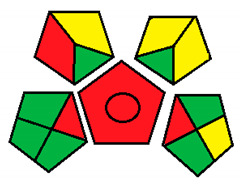
**2**	Sample preparation Collection (1) Preservation Transport (3) Storage (4) Method category: direct or indirect (5) Scale of extraction (6) Solvents/reagents used (7) Extra treatments (8) Reagent and Solvents Amount (9) Health risk (10) Safety risk (11) Instrumentation Energy consumption (12) Occupational risk (13) Waste (14) Waste treatment (15)	[35] Online (yellow) None (green) None (green) Under special conditions (green) Extraction required (red) Nano-extraction (green) Non-green solvents and reagents (red) Simple treatment (yellow) 10< mL (green) NFPA = 2, moderate toxicity (yellow) NFPA = 3, high flammability (yellow) >1.5 Kwh per sample (red) Hermetic sealing of analytical process (green) 1–10 mL (yellow) Recycling possible (green)	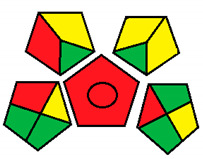
**3**	Sample preparation Collection (1) Preservation Transport (3) Storage (4) Method category: direct or indirect (5) Scale of extraction (6) Solvents/reagents used (7) Extra treatments (8) Reagent and Solvents Amount (9) Health risk (10) Safety risk (11) Instrumentation Energy consumption (12) Occupational risk (13) Waste (14) Waste treatment (15)	[36] Offline (red) None (green) Required (yellow) Samples must be refrigerated (yellow) Simple procedure (yellow) Micro-extraction (yellow) Non-green solvents and reagents used (red) None (green) 10–100 mL (yellow) NFPA = 2, Moderate toxicity (yellow) NFPA = 3, high flammability (yellow) >1.5 Kwh per sample (rrd) Hermetic sealing of analytical process (green) 10 mL (red) > Recycling possible (green)	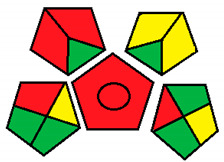
**4**	Collection (1) Preservation Transport (3) Storage (4) Method category: direct or indirect (5) Scale of extraction (6) Solvents/reagents used (7) Extra treatments (8) Reagent and Solvents Amount (9) Health risk (10) Safety risk (11) Instrumentation Energy consumption (12) Occupational risk (13) Waste (14) Waste treatment (15)	[37] Offline (red) None (green) Required (yellow) Samples must be refrigerated (yellow) Simple procedure (yellow) Micro-extraction (yellow) Non-green solvents and reagents used (red) None (green) 10–100 mL (yellow) NFPA = 2, moderate toxicity (yellow) NFPA = 3, high flammability (yellow) >1.5 Kwh per sample (red) Hermetic sealing of analytical process (green) 10 mL (yellow) 1- Recycling possible (green)	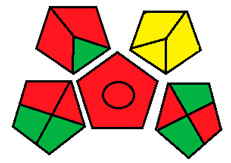
**5**	Collection (1) Preservation Transport (3) Storage (4) Method category: direct or indirect (5) Scale of extraction (6) Solvents/reagents used (7) Extra treatments (8) Reagent and Solvents Amount (9) Health risk (10) Safety risk (11) Instrumentation Energy consumption (12) Occupational risk (13) Waste (14) Waste treatment (15)	[38] Offline (red) None (green) Required (yellow) Samples must be refrigerated (yellow) Simple procedure (yellow) Micro-extraction (yellow) Non-green solvents and reagents used (red) None (green) 10–100 mL (yellow) NFPA = 2, moderate toxicity (yellow) NFPA = 3, high flammability (yellow) >1.5 Kwh per sample (red) Hermetic sealing of analytical process (green) 10 mL (red) > Recycling possible (green)	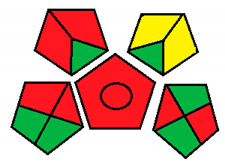
**6**	Collection (1) Preservation Transport (3) Storage (4) Method category: direct or indirect (5) Scale of extraction (6) Solvents/reagents used (7) Extra treatments (8) Reagent and Solvents Amount (9) Health risk (10) Safety risk (11) Instrumentation Energy consumption (12) Occupational risk (13) Waste (14) Waste treatment (15)	[39] Offline (red) None (green) None (green) Under special conditions (red) No sample preparation (green) Nano-extraction (green) Non-green solvents and reagents used (red) None (green) 10 mL (yellow)> NFPA = 2, moderate toxicity (yellow) NFPA = 3, high flammability (yellow) >1.5 Kwh per sample (red) Hermetic sealing of analytical process (green) −10 mL (yellow) 1 Recycling possible (green)	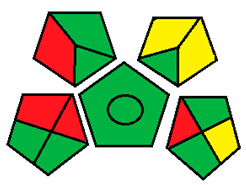
**7**	Collection (1) Preservation Transport (3) Storage (4) Method category: direct or indirect (5) Scale of extraction (6) Solvents/reagents used (7) Extra treatments (8) Reagent and Solvents Amount (9) Health risk (10) Safety risk (11) Instrumentation Energy consumption (12) Occupational risk (13) Waste (14) Waste treatment (15)	[40] Online (yellow) None (green) Required (yellow) Under special conditions (yellow) Extraction required (red) Nano-extraction (green) Non-green solvents and reagents used (red) Simple treatment (green) 10–100 mL (yellow) NFPA = 2, moderate toxicity (yellow) NFPA = 3, high flammability (yellow) >1.5 Kwh per sample (red) Hermetic sealing of analytical process (green) 10 mL (red) < Recycling possible (green)	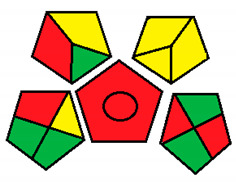

## Data Availability

Data can be provided by authors upon request.
